# Sexual Violence in Women with HIV Positive Spouse and Their Mental Health

**DOI:** 10.34172/jrhs.2020.07

**Published:** 2020-02-27

**Authors:** Saman Torkashvand, Azar Pirdehghan, Nasrin Jiriaee, Mahsan Hoseini, Mohammad Ahmadpanah

**Affiliations:** ^1^Student Research Center, Hamadan University of Medical Sciences, Hamadan, Iran; ^2^Research Center for Health Sciences, Hamadan University of Medical Sciences, Hamadan, Iran; ^3^Department of Community and Preventive Medicine, School of Medicine, Hamadan university of Medical Sciences, Hamadan, Iran; ^4^Hamadan Province Health Center, Hamadan University of Medical Sciences, Hamadan, Iran; ^5^Behavioral disorders and substances abuse research center, Hamadan University of Medical Sciences, Hamadan, Iran

**Keywords:** Sex offenses, HIV, Women’s health

## Abstract

**Background:** Sexual violence (SV) against women is one of the most important issues in women's health. We aimed to investigate the related variable for SV against women with HIV spouse and its relationship with mental problems in them.

**Study design:** A cross-sectional study.

**Methods:** This study was performed on 143 women referred to Hamadan and Malayer Risky Behavior Related Disease Clinic (Triangular Clinic), located in Comprehensive Health Service centers, Iran in 2019. SV and mental problems were assessed using standard questionnaires, based on interview. All analyses performed using SPSS. The significance level of all analyses was considered 0.05.

**Results:** Totally, 407 HIV+ patients were diagnosed from 1998 to 2018, in Hamadan and Malayer cities. We assessed the wives of survivors who allowed about SV. Mean of SV in women with HIV positive spouse was significantly higher than control group (P=0.004). Among all variables, unsafe sex, extramarital relationship, smoking, alcohol, multi-partner and suicide were significantly related to SV; but age, living area, educational status, income and job in both men and women had not statically significant relationship with SV.

**Conclusion:** SV besides its complications and mental problems in women with HIV positive spouses must be considered in interventional programs in order to improve sexual rights in this vulnerable group in the future.

## Introduction

Almost one in four women in the world experiences violence from an intimate partner in their lifetime. Thus, IPV is an important global health issue. IPV is ranked fifth in disability-adjusted life years (DALYs) in women in 2010^1^ included in various forms covering sexual ^2^.

On the other hand, a significant and complicated relationship exists between sexual violence and HIV infection^3^ and some biologic and behavioral mechanisms of sexual violence have been explained for its effects on women's health^4^.

HIV is a significant health issue in Iran. Since 1986 that the first HIV case was diagnosed in Iran, the HIV transmission pattern has changed tremendously. Until 2010, the principal form of transmission was injectable, but in the last ten years, sexually transmitted HIV has become the most common form of HIV Acquiring and the percentage of women living with HIV increased by about 30% from 2001 to 2013^5^.

One in three victims of HIV positive women suffer from a life-threatening psychological complication of violence such as anxiety, post-traumatic stress disorder (PTSD), suicide ideation, depression, and homicide ^6^, besides other sexually transmitted diseases, unwanted pregnancy, abortions, babies with low birth weight, and alcohol abuse which are more common sexual violence victims^7^.

Based on various researches, sexual abuse can cause significant reduction in level of self-care and adherence to treatment ^8^. The men who make violence against women are more inconvincible to use condom than non-violent men; also, data reveals that violent men are more tended to have multiple sex partner and drug abuse than others ^9^ considered in particular attention to physical and mental health of their wives.

Iran has a conservative norm on sexual behaviors. Because of that cultural problem which tends to ignore some sexual rights in women, HIV sexual transmission and some mental problems especially in this vulnerable group of women (wives of HIV positive men) are increasing rapidly. Despite Iran's Health Ministry polices focused on drug injection and HIV vertical transmission, sexual educations are insufficient, and patients are not honest with health workers about their sexual problem such as sexual violence ^10^. These issue points lack of comprehensive and transparent information about sexual violence status in Iran.

We aimed to investigate sexual violence frequency and its related variable and some mental problems included in stress, anxiety, and depression in woman with HIV positive spouse in Hamedan in order to plan some interventions and preventive programs for improving sexual rights and reducing sexual violence frequency and its complications in the future.

## Methods

This descriptive cross-sectional study was performed through census and non-probability convenience sampling on women with HIV-positive spouse referred to Hamadan and Malayer Risky Behavior Related Disease Clinic (Triangular Clinic) is located in Comprehensive Health Service centers. Triangular Clinic (TC) is a clinic set up by the Iranian Ministry of Health and Medical Education, besides the other units in Comprehensive Health Service Center and operates in three areas: education, counseling, medical prevention and treatment for 3 health problem included in addiction, AIDS and other STD. The services of these clinics are free of charge and provided in an anonymous manner.

Counseling Centers in year from Mar 2017 till Mar 2018. The wives of men with HIV-positive who had an active profile in the clinics aged 15 to 50 were included. They came to Hamedan and Malayer behavioral disease clinics between Mar 2017 and Mar 2018 of the, for counseling, HIV test, or being informed about their husbands' diseases.

Data were collected through a questionnaire in different parts: demographic data, checklist of risky behaviors of men extracted by the writer from the patients' profiles in the clinic, sexual violence collected by the clinical psychologist and physician through interview with women and final part contained questions on the psychological disorders, answered through the self-declaration of those who refer to clinics.

Sexual violence during the last three months was measured by the questionnaire. Its reliability was measured through Cronbach's alpha test, in which the reliability coefficient equaled to 0.88. The questionnaire consists of 17 questions classified on a five-item Likert scale (never, rarely, sometimes, often, and always). 10 of the 17 items refer to sexual violence, and 7 questions consider the resulting damages of sexual violence. In this research, 3 was considered as the cut of point ^11^. Moreover, for the frequency of psychological disorders, the DASS-21 questionnaire, which includes the three subscales of depression, stress, and anxiety, was used. This questionnaire has the capacity of measuring the three scales of depression, stress, and anxiety during the last week, and consists of 21 questions. Cronbach's alpha coefficient for each of the three factors of depression, anxiety and stress is 0.81, 0.74, and 0.78, respectively ^12^.

The checklist of risky behavior was designed by the researcher, which includes two main parts: The men part with six questions, and the women part with 17 questions.

Control group were selected for comparing and making the result more transparent. They were included in 100 women with HIV negative spouses, among those who refer to other units in Hamadan and Malayer Comprehensive Health Services (beside TC) for receiving other health services. They were group matched with the case group based on their ages. Questionnaires and interviews were similar in both groups.

The study was started after approval from institute’s Ethical Committee (ID: IR. UMSHA.REC.1396, 566). In all stages of taking information from participants and interviewing them, confidentiality was respected and they were assured .consent form was obtained at the first step of collecting data.

After collecting data, all analyses performed using SPSS version 21 (Chicago, IL, USA). Monte-Carlo test was used for investigating the relationship between qualitative variables and Mann- Whitney for comparing total score of sexual violence in two women’s groups. Spearman’s – rho was used in order to determine correlation between sexual violence score and mental problems. The significance level of all analyses was considered 0.05.

## Results

**Figure 1:**
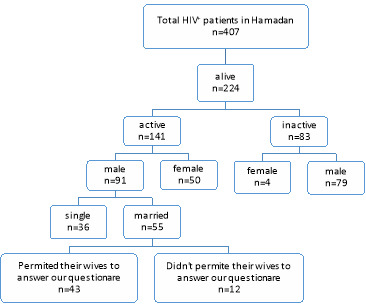
HIV+ patients in Hamadan and Malayer cities

Overall, 407 HIV^+^ patients were diagnosed from 1998 to 2018, in Hamadan Province, western Iran (Hamadan and Malayer cities). Among them 183 (44.9%) died because of different reasons and from survivors, just 141(62.9%) have followed up their disease actively and come to TC for visiting or getting their free drugs. From those 91 persons were male who their wives were wanted to answer the questions about sexual violence in our study whereas, only 43 women cooperated (Figure 1).

In order to compare the results, this survey considered 100 socio-demographic and age-matched women referred to other units of Comprehensive Health Service Center, as control group.

Of the 143 women aged 22 to 55 years (mean: 37 yr; SD: 5.9), 21.7% were from rural areas. The majority of them (72%) were housewives and more than half (56.6%) were illiterate or under diploma (Table 1).

**Table 1:** Sociodemographic characteristics of Patients Referred to the Risky Behavior Related Disease Clinic (Triangular Clinic): Iran 2019

**Variables**	**Women with HIV+ spouse , n=43**		**Women with HIV- spouse , n=100**		**HIV+ men , n=43**		**HIV- men , n=100**	
	**Number**	**Percent**	**Number**	**Percent**	**Number**	**Percent**	**Number**	**Percent**
HIV infection								
Positive	38	88.4	0	0	43	100	0	0
Negative	5	11.6	100	100	0	0	100	100
Life location								
Urban	40	93.0	67	67.0	38	88.4	72	72.0
Rural	3	7.0	33	33.0	5	11.6	28	28.0
Education								
Illiterate	6	14.0	2	2.0	5	11.6	6	6.0
Schooling	37	83.7	67	67.0	38	62.0	60	60.0
Academic	1	2.3	31	31.0	0	0	34	34.0
Job								
Housewife/unemployed	39	90.7	64	64.0	8	18.6	2	2.0
Seasonal job	1	2.3	11	11.0	22	51.2	33	33.0
Steady job	1	2.3	19	19.0	13	30.2	63	63.0
Substance abuse								
Yes	1	2.3	0	0.0	30a	69.8	2	2.0
No	42	97.7	100	100	13	30.2	98	98.0
Multi partner								
Yes	6	14.0	0	0.0	7	16.3	1	1.0
No	37	86.0	100	100	36	83.7	99	99.0
Condom use in sexual activity								
Yes	5	11.6	1	1.0	5	11.6	1	1.0
No	38	88.4	99	99	38	88.4	99	99.0
Unwanted pregnancy								
Yes	10	23.3	21	21.0	-	-	-	-
No	33	76.7	79	79.0	-	-	-	-
History of suicide								
Yes	3	7.0	0	0.0	-	-	-	-
No	40	93.0	100	100	-	-	-	-
Other sexual transmitted diseases								
Yes	10	23.3	0	0.0	-	-	-	-
No	33	76.7	100	100	-	-	-	-

Totally, sexual violence mean in women with HIV positive spouse was 6.7 ±5.7 and in control group was 3.8 ±3.6 (*P* =0.004). Among all questions, frequency of “forced to see pornographic pictures or videos” in those vulnerable women in comparison with controls was 41.9% vs. 6.0%, about “ignoring the woman sexual pleasure” it was 67.5% vs. 25% and for “Ignoring privacy in Sex” it was 44.2% vs. 7.0% that all of them were significantly different (Table 2).

**Table 2:** Sexual violence against Women with HIV positive spouse and control group

**Questions**	**Women with HIV+ spouse**		**Control group**		***P*** **value**
	**Number**	**Percent**	**Number**	**Percent**	
1. Sexual relationship, regardless of woman satisfaction					0.335
Never	24	55.5	42	42.4	
Sometimes	17	39.5	48	48.5	
Always	2	4.7	9	9.1	
2. Sex with force					0.404
Never	36	83.7	85	85	
Sometimes	5	11.6	14	14	
Always	2	4.7	1	1	
3. Unusual sex( anal, oral)					0.051
Never	30	69.8	79	80.6	
Sometimes	11	25.6	19	19.4	
Always	2	4.7	0	0	
4.lack of sex in order to punishment					0.116
Never	37	86	94	94	
Sometimes	6	14	6	6	
Always	0	0	0	0	
5. Forced to have sex without contraceptives					0.062
Never	25	62.5	39	42.9	
Sometimes	5	12.5	27	29.7	
Always	10	25	25	27.5	
6. Forced to see pornographic pictures or videos					0.001
Never	25	58.1	94	94	
Sometimes	16	37.2	6	6	
Always	2	4.7	0	0	
7. Ignoring the woman sexual pleasure					0.001
Never	14	32.6	75	75	
Sometimes	18	41.9	22	22	
Always	11	25.6	3	3	
8. Ignoring privacy in Sex					0.001
Never	24	55.8	93	93	
Sometimes	5	11.6	5	5	
Always	14	32.6	2	2	
9. Physical injury during sex (biting, kicking, slap or….)					0.473
Never	29	67.4	76	76	
Sometimes	12	27.9	22	22	
Always	2	4.7	2	2	
10. Threatening woman and her children if she avoid sex					0.823
Never	42	97.7	97	97	
Sometimes	1	2.3	3	3	
Always	0	0	0	0	

In recent study, several men and women demographic and risky behavioral variables related to sexual violence were checked. Among all those variables, age, living area (rural or urban), educational status, income and job in both men and women had not statically significant relationship with sexual violence (*P* >0.050). The relationship between sexual violence and other variables categorized to risky behaviors of men and women and some conditions resulted from sexual violence are shown in Table 3. Additionally, correlation between sexual violence and age of marriage in participants was statically significant (Spearman’s – rho=-0.245; *P* =0.005).

**Table 3:** Comparing the of sexual violence in terms of demographic and risky behaviors of women and their spouse

**Variables**	**Sexual violence score**		***P*** **value**
	**Mean**	**SD**	
Risky behaviors of men			
Substance abuse			0.066
No	4.2	4.3	
Yes	6.1	5.0	
Unsafe sex			0.013
No	4.5	4.1	
Yes	8.5	6.3	
Extramarital relationship			0.030
No	4.3	4.1	
Yes	9.6	7.1	
Risky behaviors of women			
Smoking			0.027
No	4.5	4.0	
Yes	11.0	5.0	
Alcohol			0.029
No	4.5	4.0	
Yes	11.3	4.5	
Multi partner			0.012
No	4.5	4.2	
Yes	10.2	5.2	
Conditions related to sexual violence			
HIV in women			0.001
Positive	5.9	0.9	
Negative	3.7	0.3	
Abortion related to sexual violence			0.007
No	4.5	4.0	
Yes	16.0	5.6	
Suicide attempt			0.030
No	4.5	4.4	
Yes	11.0	5.5	
Sexual transmitted diseases			0.023
No	4.4	4.0	
Yes	9.7	6.4	

**Figure 2:**
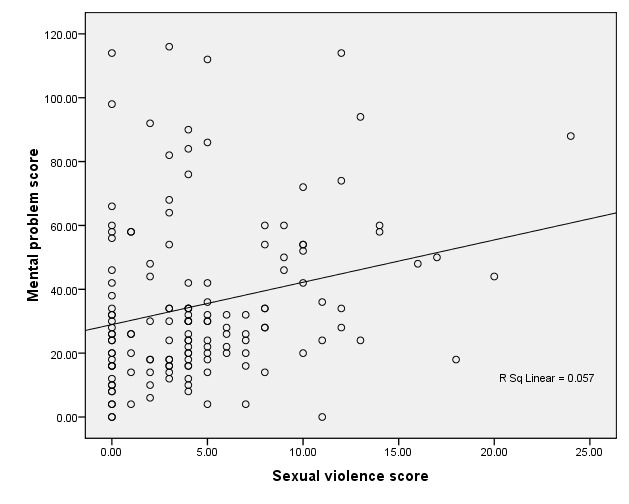
Correlation between sexual violence and mental problems

In order to investigate sexual violence and mental problems correlation, depression anxiety and stress were considered separately (Table 4) and totally (Figure 2). There was statically significant relationship between sexual violence score and mental problems (spearman’s – rho=0.249; *P* =0.003).

**Table 4:** Correlation between sexual violence score based on Spearman’s – rho correlation and mental problems

**Variables**	**rho**	***P*** **-value**
Depression	0.242	0.004
Anxiety	0.197	0.018
Stress	0.217	0.009

However in separate analysis, there was not significant correlation between SV and depression (Spearman’s – rho=0.036; *P* =0.819), anxiety (Spearman’s – rho=0.028; *P* =0.859) and stress (Spearman’s – rho=0.126; *P* =0.419) in women with HIV positive spouse. In control group there was significant correlation between SV and depression (Spearman’s – rho=0.238; *P* =0.017) and SV and stress (Spearman’s – rho=0.237; *P* =0.018) but correlation between SV and anxiety was not significant (Spearman’s – rho=0.196; *P* =0.051).

Among all 143 assessed women 12 (8.3%) had experienced at least one type of sexual abuse during childhood. The sexual abuse experience frequency in Women with HIV positive spouses was 23.2% (10 person), whereas it was 2% (2 person) in control group. Therefore, there was statically significant difference in childhood sexual abuse between these two groups (Monte Carlo: *P* <0.001).

## Discussion

Over the past 20 years, 407 HIV+ people have been identified in Hamadan and Malayer; out of this population, 22.3% had an active medical record, and among them, 88% permitted participation of their spouses in the study.

Regarding the high-risk behaviors, 35.67% of HIV+ men studied in this research were an injection drug abuser. In a similar study investigating sexual violence imposed on women by HIV+ men in Russia, 50.1% of the men had a drug abuse background ^13^. In Iran, 61.5% of HIV+ people were injection drug abuse^14^; this finding was similar to the result of our research.

In this study, only 12% of the studied couples regularly used a condom; this percentage is significant compared with the value reported in the control group (1%). However, it is not an optimal rate. According to national HIV guidelines ^15^, all the couples’ one or both of affected by HIV should permanently use a condom; because in every unprotected sexual intercourse, the virus can become transferred from the person with a higher viral load to the partner with a lower viral load. In this study, 88% of the population members did not follow these instructions; whereas in the study investigating the sex workers in Turkey^16^, 70% of the subjects always used a condom and 29.2% of them usually used a condom. In another study performed in Iran, only 17.2% of women with high-risk behaviors used a condom^17^.

Meanwhile, significant correlation was found between high-risk behaviors such as unsafe sex and extramarital relationship in men and sexual violence against their wives. However, contrary to many studies, this association was not significant with addiction. The insignificant correlation between addiction level and sexual violence can be justified based on the theory that studies performed in other parts of the world have reported the use of cocaine that is a CNS stimulant. For example, 50.0% of men imposing violence on their wives reported the use of cocaine ^18^. However, the majority of the addicted men studied in this research reported injection addiction to heroin which is CNS depressant. Regarding the high-risk behaviors in HIV+ women, the results of this study are consistent with Bonner’s findings ^19^; there is a correlation between sexual violence and increased alcohol consumption in the victimized women; these phenomena are related to increasing of HIV acquiring. A study suggested a significant correlation between suicide attempts and sexual violence ^20^.

Various studies have investigated the occurrence of sexual violence in different parts of the world. The frequency of sexual violence cases in India, Zimbabwe, and Ethiopia has been respectively reported as 9%, 14%, and 49.1%. Unlike the results of this study and the study performed in India ^21^, the two studies performed in African countries^22,23^ did not reveal any significant difference between sexual violence rates in HIV+ people and the control group subjects. These differences between the results of these studies are due to the cultural differences in the studied countries. In Africa, due to the epidemic status of sexual violence and poverty, women’s HIV condition does not seem to be a determinative factor of exposure to sexual violence ^3^. However, due to the lower rates of violence in Iran and India, HIV+ is a determinative factor of exposure to violence. The results of other studies suggest that sexual violence can significantly increase the risk of HIV^24^.

In Iran, few reliable researches have investigated sexual violence in HIV+ women; however, in studies investigating other women, 47.3% of infertile women in Tehran and 9.7% of married women in the East of Iran exposed to sexual violence^25,26^.

This paper investigated the correlation between various factors and sexual violence based on demographic information and risk factors in women and their HIV+ husbands.

Unsafe sex and extramarital relationship rates were significantly higher in men imposing sexual violence. However, there was no correlation between sexual violence and other factors, such as education level, income, and substance abuse. In women, sexual violence related to risk factors such as smoking, alcohol consumption, and multi-partner. In this study, a significant correlation was found between sexual violence-related abortion, STD, and suicide attempts and sexual violence in women.

In present study, significant association between sexual violence and depression, stress, and anxiety were shown; the most significant correlation is between sexual violence and depression^27^. In another study the relation between violence, HIV, substance abuse, and their effect on depression was investigated in women, there were higher rates of violence, substance abuse, and depression in women affected by HIV than HIV- women. Among HIV+ women, there was a significant correlation between depression and substance abuse ^28^. These findings are consistent with the results of our study.

In this research, a significant difference was found between the experience of sexual violence in childhood in women with HIV+ husbands and the control group. In Iran in 2013, a correlation was found between child sexual abuse, parents’ substance abuse, and low socioeconomic level^29^.

However our research had some limitations. First one was statistical constraints regarding the few numbers of samples. The second limitation was that 12 HIV+ men did not agree with the participation of their wives in the study. These men were among the people imposing violence. Therefore, the violence rate evaluated in this study has been probably estimated less than the real value. The next, in the research process, a group of the population members were not quite aware of their sexual rights, and they had not received any education in this area. In this research, they became familiar with the concept of sexual violence for the first time. Unfortunately, previous reliable studies on sexual violence in women with HIV+ husbands and the association between violence, alcohol consumption, and substance abuse were not found. Therefore, this research can consider as pioneer in this regard.

Future studies focusing on women’s knowledge and attitude towards sex and providing preventive and promoting sexual literacy programs for the affected women are necessary.

## Conclusion

The sexual violence rate in women with HIV is significantly higher than women not affected by this virus. Furthermore, people with higher rates of violence report more high-risk behaviors such as alcohol consumption, smoking, and multi-partner.

## Acknowledgements

We gratefully acknowledge all managers and staff in Hamadan and Malayer Risky Behavior Related Disease Clinic (Triangular Clinic) that helped for data collection and sensitive interview with participants.

## Conflict of interest

The authors declare that they have no conflict of interests.

## Funding

This research has been funded by Hamadan University of Medical Sciences.

## Highlights

- Sexual violence against women with HIV^+^spouse was higher than controls.
- Abortion, STD and suicide were correlated with sexual violence in women.
- There was an association between sexual violence and depression, stress and anxiety.
